# A pilot study comparing bovine mesenteric artery and expanded polytetrafluoroethylene grafts as non-autogenous hemodialysis options

**DOI:** 10.1590/1677-5449.007117

**Published:** 2018

**Authors:** Bruno Morisson, Antonio Luiz de Araújo, Leonardo de Oliveira Harduin, Eglina Filgueiras Porcari, Rossano Kepler Alvim Fiorelli, Stenio Karlos Alvim Fiorelli, Jose Marcos Braz Serafim, Julio Cesar Peclat de Oliveira

**Affiliations:** 1 Universidade Federal do Estado do Rio de Janeiro – UNIRIO, Departamento de Pós graduação, Rio de Janeiro, RJ, Brasil.; 2 Hospital Federal do Andarai – HFA, Departamento de Cirurgia Vascular, Rio de Janeiro, RJ, Brasil.; 3 Sociedade Brasileira de Angiologia e de Cirurgia Vascular – SBACV, São Paulo, SP, Brasil.; 4 Universidade Federal do Estado do Rio de Janeiro – UNIRIO, Departamento de Cirurgia Vascular, Rio de Janeiro, RJ, Brasil.

**Keywords:** fistula, hemodialysis, patency

## Abstract

**Background:**

Many dialysis patients do not have the necessary conditions for construction of a native arteriovenous fistula (AVF). Expanded Polytetrafluoroethylene (ePTFE) vascular prostheses are the most widely-used option, but it is known that they are inferior to native vein AVFs.

**Objectives:**

To identify a graft with superior performance to ePTFE, comparing their results with those of AVFs made from bovine mesenteric arteries treated with L-Hydro technology (Labcor Laboratories ^®^).

**Methods:**

A prospective and controlled study of 10 patients with AVFs constructed with ePTFE and 10 patients with L-Hydro bioprostheses, matched for comorbidities. The variables studied were: primary patency, assisted primary patency, and secondary patency, surgical manipulability, and prevalence of infections. The performance of prostheses was assessed by duplex-scan and repeated consultations with health professionals at hemodialysis clinics. The chi-square test was used for statistical analysis.

**Results:**

After 1 year of postoperative follow-up, secondary and primary patency rates were higher for L-Hydro than ePTFE AVFs. Fewer interventions were needed to maintain AVF patency in the L-Hydro AVF group. The most common complication was graft thrombosis, which was more frequent in the ePTFE group. While the figures indicate more favorable outcomes in the L-Hydro AVFs, this could not be confirmed with the statistical treatment employed.

**Conclusions:**

The L-Hydro graft appears to be a valuable alternative option for AVFs, since it seems to require fewer interventions to maintain patency when compared to ePTFE grafts.

## INTRODUCTION

 Arteriovenous fistulas (AVFs) constructed using the native veins of the patient’s forearm are recognized as the best option for definitive hemodialysis vascular access. Sadly, in many patients, because of the comorbidities frequently present in patients with chronic renal failure (such as diabetes mellitus, connective tissue diseases, and systemic arterial hypertension) excessive manipulation of the native veins of the forearm makes them useless for construction of AVFs. 1

 Expanded polytetrafluoroethylene (ePTFE) vascular prostheses are the most widely-used alternative for construction of AVFs in cases in which there are no native veins available in the forearm, but their use is associated with elevated rates of infectious complications and premature graft occlusions. 

 in the 1970s, vascular bioprostheses constructed from bovine pericardium preserved in glutaraldehyde were widely employed in attempts to identify a vascular substitute that offers superior performance to ePTFE for construction of AVFs for hemodialysis. 2 However, postoperative follow-up of the performance of these grafts was beset by complications affecting significant numbers of patients (neointimal hyperplasia and degeneration of the biological tissue), 3 so they were abandoned for construction of hemodialysis AVFs. Degeneration of the bovine pericardium used for construction of the vascular bioprostheses was attributed to immunoresponses provoked by graft-host reactions, since preserving biological tissues with glutaraldehyde does not entirely eliminate the xenograft’s antigenicity. Another factor identified as responsible for the adverse results of bovine pericardium vascular bioprostheses is glutaraldehyde’s intrinsic cytotoxicity, which impedes endothelization of the internal surface of the vascular bioprosthesis. 

 Development of endovascular techniques for treatment of stenosis in arteries and veins has revived interest in using biological vascular grafts for construction of AVFs. This is because any stenoses of grafts or anastomoses that emerged during the postoperative period could be treated using endovascular procedures, resulting in greater AVFs durability. 

 Vascular bioprostheses preserved in L-Hydro (Labcor Laboratories^®^) are the result of application of a preservation process known as L-Hydro to bovine mesenteric arteries. L-Hydro technology enables antigens and cellular components to be extracted from the biological tissue, while preserving essential extracellular elements, such as elastin and collagen. The biological behavior of vascular bioprostheses preserved with L-Hydro technology shares the autologous vein’s capacity to achieve full endothelization. Graft healing is completed by incorporation of myofibroblasts into the collagen and elastin framework in the tunica media of the bovine mesenteric artery. 4 ^,^ 5

 The objective of this study was to compare the performance of AVFs constructed using ePTE vascular prostheses with AVFs constructed using vascular bioprostheses preserved with L-Hydro technology, according to the following parameters: primary patency, assisted primary patency and secondary patency; surgical handling properties of the vascular prostheses used to construct AVFs; and complications related to the vascular prostheses used to construct AVFs. 

## METHOD

 The research protocol was assessed by the institution’s Ethics Committee and registered on the Plataforma Brasil (number: 46166115.4.0000.5258). All patients were duly informed of the proposal for their voluntary enrollment on the study and signed consent forms prior to preoperative assessment. 

 The study inclusion criteria were: adult dialysis patients with chronic renal failure who did not have native veins in their upper limbs adequate for construction of AVFs for hemodialysis; brachial artery diameter > 3 mm at the cubital fossa and axillary vein diameter > 3 mm at its most distal point (according to the Kidney Diseases Outcomes Quality Initiative, KDOQI, recommendations), measured preoperatively by duplex scan. 

 Patients were allocated at random to one of two groups: the ePTFE AVF group, comprising 10 patients who had AVFs constructed with ePTFE vascular prostheses (FlowLine Bipore vascular grafts, JOTEC^®^); or the L-Hydro AVF group, comprising 10 patients who had AVFs constructed using vascular bioprostheses preserved with L-Hydro technology (Labcor Laboratories ^®^). 

 Patients were prospectively enrolled on the study at random and alternately into each of the experimental groups, from August 1, 2013, to December 31, 2015. A total of 20 AVFs were constructed at the Hospital Federal do Andaraí (HFA). 

**Figure 1:**
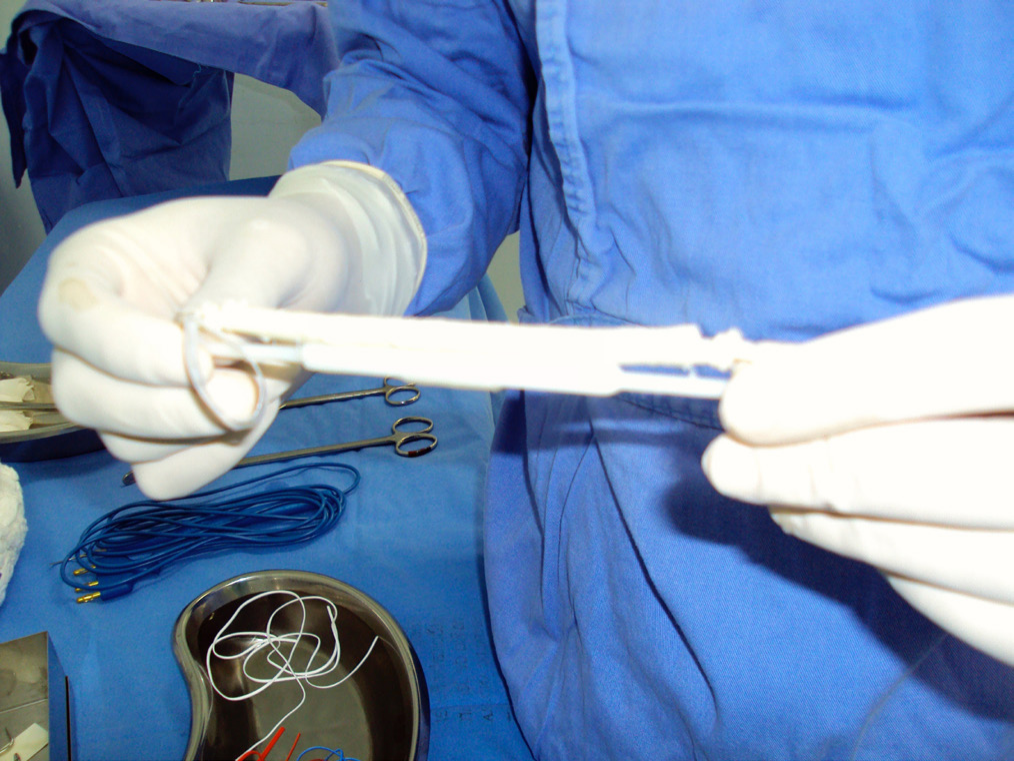
Prosthesis before implantation.

**Figure 2:**
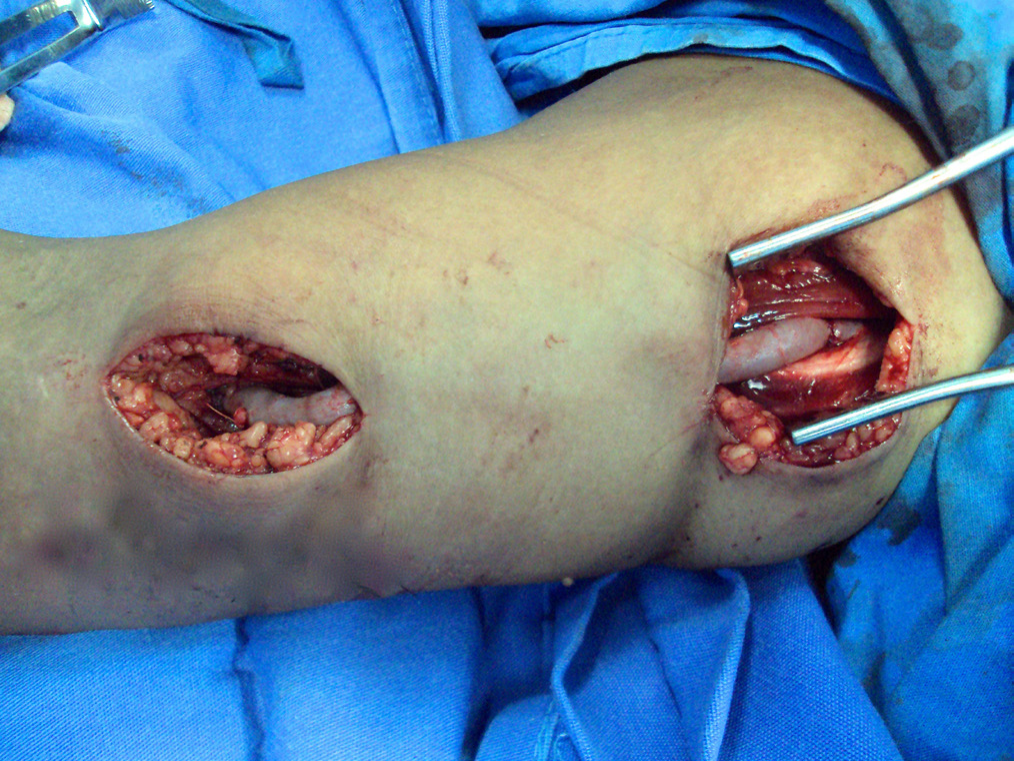
Prosthesis in place.

 Surgical procedures were conducted with anesthetic block at the brachial plexus and sedation. In all patients, AVFs were constructed with systemic heparinization and the vascular prosthesis (whether ePTFE or L-Hydro) was implanted within the subcutaneous plane under the anterolateral surface of the arm (within the topography of the biceps muscle). Proximal anastomosis with the brachial artery was performed at the level of the cubital fossa, and the distal anastomosis was with the most distal portion of the axillary vein. In the L-Hydro AVF group, anastomosis to the brachial artery was performed before anastomosis to the axillary vein. The bioprosthesis was filled with arterial blood under pressure, guided through the subcutaneous tunnel, and brought out at the point where the axillary vein was dissected. This strategy was intended to prevent torsion and kinking along the bioprosthesis’ path through the subcutaneous tunnel. All patients were examined as soon as possible after the operation (while still in the post-anesthetic recovery room) for perfusion distal to the AVFs (capillary refill time and ipsilateral hand strength) by the surgeon responsible for constructing the AVFs. 6 The study’s postoperative follow-up period was 2 years. The postoperative follow-up protocol was based on criteria established by KDOQI 7 ( Figures 1 and 2 ). 

**Figure 3:**
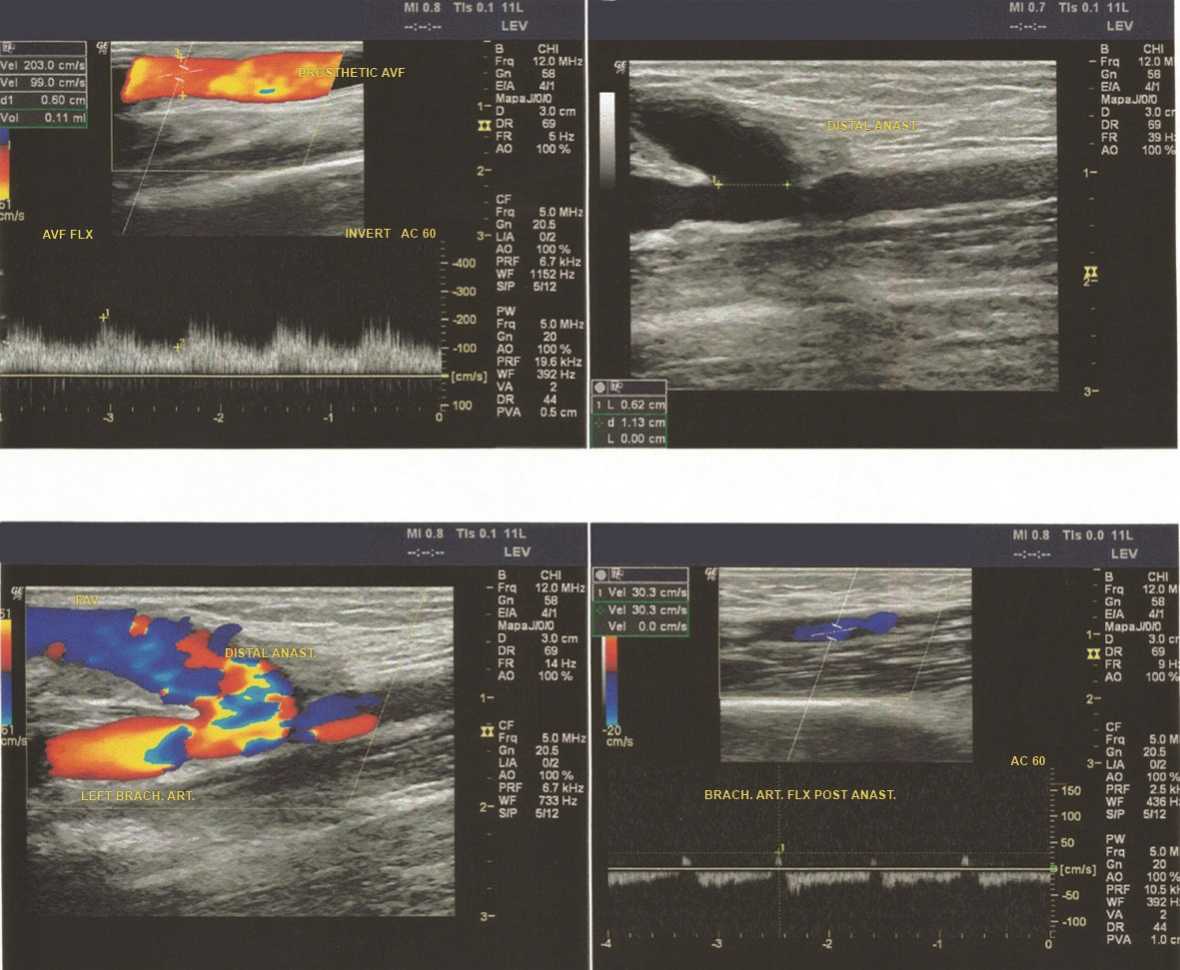
Control duplex scan.

 All patients were assessed during postoperative follow-up using Doppler vascular ultrasonography to scan AVFs for stenosis or dilatation of the vascular prosthesis; changes in the thickness of the vascular prosthesis wall; stenosis at anastomosis sites (neointimal hyperplasia); and presence of hematoma or periprosthetic accumulations; and to estimate AVF flow rate; 8 and evaluate the response to antibiotic therapy in cases of infected prostheses ( Figure 3 ). 

 Clinical criteria related to AVF performance were also evaluated during hemodialysis sessions. These included presence of pulse waves along the AVF path; presence of continuous thrill along the AVF path; difficulties with puncture of the prosthesis; detection of persistent bleeding at the puncture site after needle removal; and need for dialysis machine parameters to be reprogrammed in order to achieve effective dialysis. 8

### Acute AVF occlusion

 In cases of clinical suspicion of AVF occlusion (disappearance of thrill), the study protocol’s recommendation was immediate assessment by the surgeon and investigation with duplex scan or angiography. After confirmation of a diagnosis, therapeutic intervention with endovascular procedures was the preferred management option. Open surgery was only resorted to if endovascular treatment was impossible or failed. 

### Infection of AVFs

 A diagnosis of AVF infection was made on the basis of clinical signs (erythema, edema and pain along the prosthesis path) and confirmed by blood culture or cultures of secretions. The initial treatment proposed for infection of AVFs with prostheses was prolonged antibiotic therapy. Surgical removal of the vascular prosthesis was indicated if treatment with antibiotics was unsuccessful. 9

### Flow steal syndrome

 Steal syndrome was defined using the ischemia stages classification: 9 ^,^ 10

- Stage I: pale-blue and/or cold hand, with no pain;
- Stage II: pain triggered by exercise and/or during hemodialysis;
- Stage III: pain at rest in the ipsilateral hand;
- Stage IV: ulcers/necrosis/gangrene of the hand.

Surgical intervention would be deemed necessary in the event of Stage II or IV steal syndrome. 

### Technique for prosthesis puncture for hemodialysis

 The puncture technique employed to initiate hemodialysis with L-Hydro AVFs was the same that is used for AVFs constructed from native veins: the first access is acquired using 17G puncture needles at an angle of 25 to 30º. For subsequent hemodialysis sessions, when the skin at the puncture sites was thicker, the L-Hydro AVFs were punctured with larger-caliber needles (15G). The prosthesis puncture technique for ePTFE AVFs used a 15G caliber needle from the first hemodialysis access on. 1

The following definitions were used in this study:

- AVF primary patency: defined as the time elapsed between construction of the AVF and diagnosis of AVF dysfunction or complete occlusion of the prosthesis (thrombosis);
- Assisted AVF primary patency: defined as the duration of usability of AVFs for which there were interventions (endovascular/surgery) to correct dysfunctions diagnosed in postoperative follow-up;
- AVF secondary patency: defined as the duration of usability of AVFs for which interventions (endovascular/surgery) were conducted after complete occlusion of the prosthesis (thrombosis).

The chi-square test was used to compare rates of complications observed during the study. The significance level of statistical probability used in this study was 0.05. 

## RESULTS

 Demographic data for the patients in the L-Hydro AVF and ePTFE AVF groups were similar. With relation to frequencies of comorbidities, diagnoses of systemic and intraoperative arterial hypertension were more common in one of the study groups (L-Hydro). 

 During postoperative follow-up, there was a difference between the groups of one additional case in the ePTFE AVF group, with six interventions in three patients, whereas in the L-Hydro AVF group there were four interventions in two patients. 

 With relation to assisted primary patency, the data show that the fistula was functioning in 50% of the patients in the L-Hydro AVF group at 1-year follow-up and in 25% after 2 years of postoperative follow-up. In the ePTFE AVF group, 25% of the patients had functioning AVFs at 1-year follow-up and after 2 years just 10% of the patients had a functioning AVF (p < 0.05). 

 After 2 years’ follow-up, primary patency was 70%, in the L-Hydro AVF group and just 20% in the ePTFE AVF group (p < 0.05). 

 The analysis of AVF secondary patency did not detect statistical differences between the L-Hydro AVF and ePTFE AVF groups (secondary patency rates after 2 years’ follow-up were 60% and 50%, respectively). In this study population, the number of interventions needed to extend the service life of AVFs was 1.5 interventions/year in the L-Hydro AVF group and two interventions/year in the ePTFE AVF group. Angioplasty was the procedure most frequently employed to preserve AVF function. 

**Figure 4:**
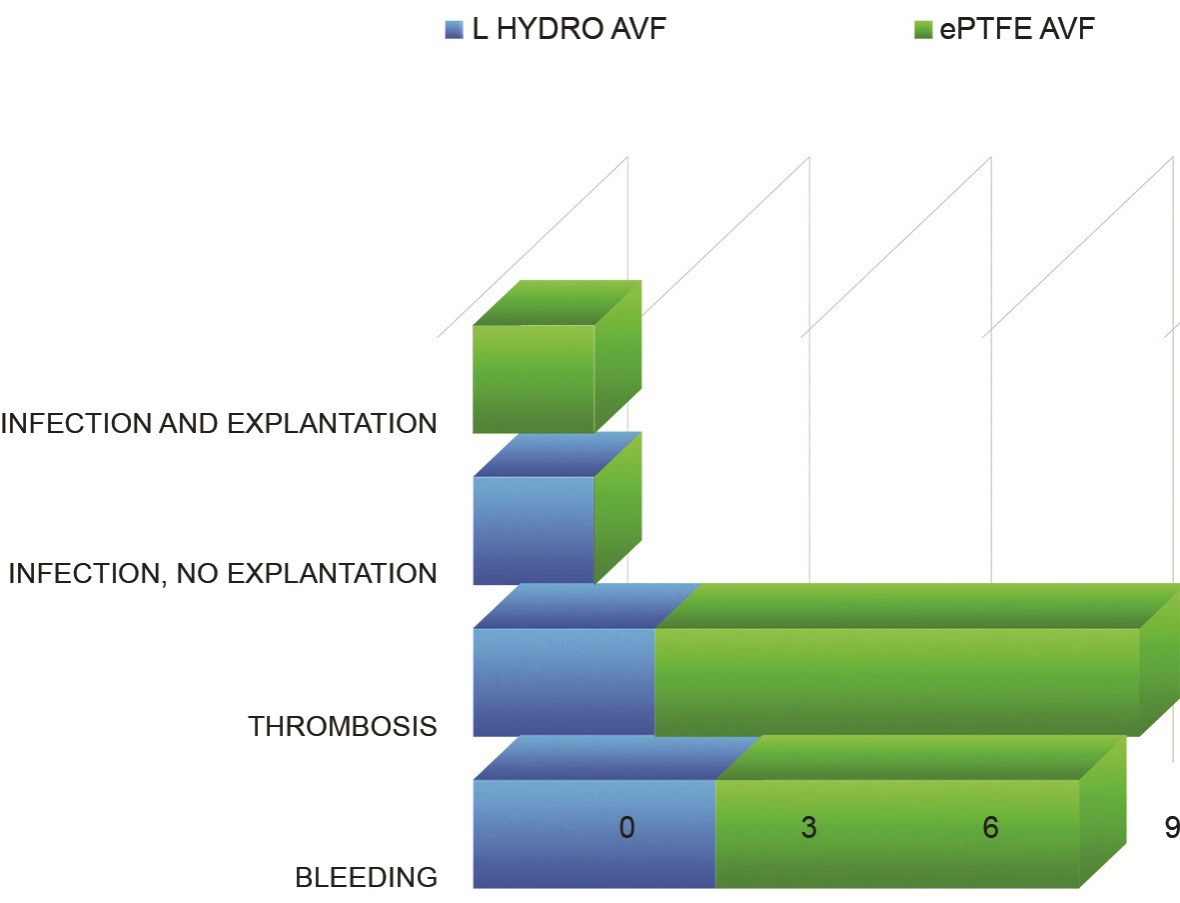
Complications.

 The most frequently-observed complications related to vascular prostheses were thrombosis, infection, and pseudoaneurysm. The most frequent complication was graft thrombosis, observed in eight patients in the ePTFE AVF group and three in the L-Hydro AVF group (p = 0.01). The frequency of graft infection was similar in both study groups, but management of the L-Hydro AVF group differed from that of the ePTFE AVF group, since in the L-Hydro AVF group prolonged treatment with antibiotics was effective for averting the need for explantation of the prosthesis ( Figure 4 ). 

 The frequencies of diagnoses of other complications, such as pseudoaneurysm formation and steal syndrome, did not exhibit statistical differences between the two study groups. 

 In one patient in the L-Hydro AVF group, with venous hypertension secondary to stenosis of a central vein, an L-Hydro bioprosthesis developed significant dilatation, causing deactivation of the AVF and removal of the graft. Anatomopathological examination of the prosthesis after removal showed that the biological tissue was intact, with endothelial lining preserved, and free from signs of degeneration. 

## DISCUSSION

 Although the number of patients was limited, in our study we observed greater primary patency among AVFs constructed using the L-Hydro bioprosthesis than for AVFs constructed with ePTFE prostheses. 11 We also observed that the L-Hydro AVF group required a smaller number of interventions for maintenance of AVF viability. The reduced need for interventions in the L-Hydro group had an impact on the lower rate of complications observed in the group. 12

 The concept of AVF primary patency is related to the time elapsed between construction of the fistula and diagnosis of dysfunction (or occlusion). A diagnosis of AVF dysfunction is generally suspected on the basis of clinical changes, observed by a technician or nurse, during the hemodialysis sessions. Perception of these signs and symptoms is dependent on the expertise and experience of the hemodialysis professionals who, ultimately, are responsible for referring the patient for assessment by the vascular surgeon. 

 Since AVF primary patency depends on a diagnosis of AVF dysfunction, which in turn cannot be standardized across all patients, it is clear that use of the primary patency variable for the purpose of comparing AVF performance is compromised. We therefore consider that the parameters assisted primary patency and secondary patency offer a firmer foundation for comparisons of AVF performance between the groups in our study. 13

 We consider that the assisted primary patency rates in the ePTFE AVF group observed in our study 14 were low (25% of the patients had functioning AVFs after 1 year of follow-up), although several prospective studies report assisted primary patency rates ranging from 10 to 43%. Two recent prospective studies of AVFs constructed with ePTFE grafts 15 also reported widely discrepant results. A retrospective study comparing the performance of AVFs constructed with ePTFE and standard PTFE 16 reported assisted primary patency rates of 35% and 25%, respectively. 

 Notwithstanding the variability of the results of comparative studies of the patency of AVFs constructed using prostheses, it is important to emphasize the result observed in our study, where the assisted primary patency rate was 70% in the L-Hydro AVF group. The small number of patients enrolled in the sample could have affected this result, but it is nevertheless a rate that is very much higher than the results of the majority of published prospective studies on assisted primary patency in AVFs constructed using ePTFE. 

 Our initial experience with vascular bioprostheses preserved in L-Hydro began in 2011, for revascularization of the lower limbs of patients without available native veins as an alternative to ePTFE vascular prostheses. We observed reduced inflammatory response at the site of bioprosthesis implantation and lower rates of infectious complications, in addition to better surgical graft malleability, when compared to ePTFE vascular prostheses. After mean postoperative follow-up of 15 months, this group of patients had a significantly lower incidence of graft thrombosis than those with ePTFE vascular prostheses. 17

 Neointimal hyperplasia frequently develops in vascular anastomoses and causes progressive graft stenosis and occlusion. A common cause of arteriovenous graft thrombosis is neointimal hyperplasia developing at anastomosis sites. The cause of neointimal hyperplasia is multifactorial mechanical stress provoked by blood turbulence in the region of the anastomosis, because of differences in the complacency of the artery wall and the wall of the vascular substitute and the shear stress resulting from the changing blood flow in the area of vascular anastomosis. The greater complacency and malleability of vascular bioprostheses preserved in L-Hydro is evidence that their use for construction of AVFs involves reduced neointimal hyperplasia-generating stimulation, when compared with ePTFE vascular prostheses. 

## CONCLUSIONS

 Bovine mesenteric artery grafts preserved in L-Hydro are an excellent option for hemodialysis. Further studies with larger numbers of participants and longer postoperative follow-up are needed to consolidate our results. 
