# Supplementary material for: A pilot study comparing bovine mesenteric artery and expanded polytetrafluoroethylene grafts as non-autogenous hemodialysis options
Source: J Vasc Bras. 2018 Oct-Dec;17(4):303–9. doi: 10.1590/1677-5449.007117 (PMC6375270; doi:10.1590/1677-5449.007117)
Supplement: Translation

# Translation: Estudo piloto comparando artéria mesentérica bovina e enxertos de politetrafluoroetileno expandido como acessos de hemodiálise não autógenos

**Authors:** Bruno Morisson, Antonio Luiz de Araújo, Leonardo de Oliveira Harduin, Eglina Filgueiras Porcari, Rossano Kepler Alvim Fiorelli, Stenio Karlos Alvim Fiorelli, Jose Marcos Braz Serafim, Julio Cesar Peclat de Oliveira

**Affiliations:** 1 Universidade Federal do Estado do Rio de Janeiro – UNIRIO, Departamento de Pós graduação, Rio de Janeiro, RJ, Brasil.; 2 Hospital Federal do Andarai – HFA, Departamento de Cirurgia Vascular, Rio de Janeiro, RJ, Brasil.; 3 Sociedade Brasileira de Angiologia e de Cirurgia Vascular – SBACV, São Paulo, SP, Brasil.; 4 Universidade Federal do Estado do Rio de Janeiro – UNIRIO, Departamento de Cirurgia Vascular, Rio de Janeiro, RJ, Brasil.

**Keywords:** fístula, hemodiálise, perviedade

## Abstract

**Contexto:**

Muitos pacientes dialíticos apresentam condições desfavoráveis para confecção de fístula arteriovenosa (FAV) nativa. A prótese vascular de politetrafluoroetileno expandido (ePTFE) é a alternativa mais utilizada, porém, sabidamente inferior àquela com veias nativas.

**Objetivos:**

Pesquisar um enxerto de performance superior à do ePTFE, confrontrando seus resultados com os de FAVs confeccionadas com artéria mesentérica bovina tratada com tecnologia L-Hydro (Labcor Laboratórios^®^).

**Métodos:**

Estudo prospectivo e controlado, composto pelo grupo controle de 10 pacientes submetidos à confecção de FAV com ePTFE (FAV ePTFE) e grupo experimental de 10 pacientes com bioprótese L-Hydro (FAV L-Hydro). Os componentes foram pareados em relação às comorbidades apresentadas. As variáveis estudadas foram: perviedades primária, primária assistida e secundária, manuseabilidade e prevalência de infecções. A performance das próteses foi avaliada por *duplex scan* e por consultas seriadas realizadas por profissionais de clínicas de hemodiálise. O tratamento estatístico foi o teste do qui-quadrado.

**Resultados:**

Após 1 ano de seguimento pós-operatório, as taxas de perviedade secundária e primária assistida foram maiores no grupo FAV L-Hydro do que no FAV ePTFE. As intervenções para manter a perviedade da FAV foram menores no grupo FAV L-Hydro. A complicação mais comum foi trombose do enxerto, mais frequente no grupo FAV ePTFE. Apesar de os números indicarem desfechos mais favoráveis nas FAV L-Hydro, não foi possível confirmar esse achado com o tratamento estatístico aplicado.

**Conclusões:**

O enxerto L-Hydro parece ser uma alternativa valiosa para FAV, pois parece necessitar de menos intervenções para manutenção da perviedade, quando comparado ao enxerto de ePTFE.

## INTRODUÇÃO

 As fístulas arteriovenosas (FAV) confeccionadas com veias nativas do antebraço são reconhecidas como a melhor alternativa de acesso vascular definitivo para hemodiálise. Infelizmente, em muitos pacientes, a manipulação excessiva das veias nativas do antebraço, em função de comorbidades frequentemente presentes em pacientes portadores de insuficiência renal crônica (tais como diabetes melito, doenças do colágeno, hipertensão arterial sistêmica), torna impossível sua utilização na confecção de FAV 1 . 

 A prótese vascular de politetrafluoroetileno expandido (ePTFE) é o substituto vascular alternativo mais utilizado para confecção de FAV quando não existem veias nativas disponíveis no antebraço, a despeito do número elevado de complicações infecciosas e oclusões precoces do enxerto associados à sua utilização. 

 Na busca de um substituto vascular com performance superior à da ePTFE para confecção de FAV para hemodiálise, foram amplamente utilizadas, na década de 1970, biopróteses vasculares de pericárdio bovino preservadas em glutaraldeído 2 . O acompanhamento pós-operatório da performance desses enxertos foi marcado por complicações – hiperplasia neointimal e degeneração do tecido biológico – em número significativo de pacientes 3 , levando ao abandono de seu uso na confecção de FAV para hemodiálise. A degeneração do pericárdio bovino utilizado na confecção da bioprótese vascular foi atribuída à resposta imune provocada pela reação enxerto-hospedeiro, uma vez que a preservação de tecidos biológicos com glutaraldeído não elimina totalmente a antigenicidade do xenoenxerto. Outro fator apontado como responsável pelos resultados adversos da bioprótese vascular de pericárdio bovino é a citotoxicidade intrínseca do glutaraldeído, que impede a endotelização da superfície interna da bioprótese vascular. 

 O desenvolvimento de técnicas endovasculares para tratamento de estenoses em artérias e veias reavivou o interesse na utilização de enxertos vasculares biológicos para confecção de FAV. As eventuais estenoses no enxerto ou nas anastomoses, surgidas no pós-operatório, poderiam ser tratadas por procedimento endovascular, resultando em maior durabilidade da FAV. 

 A bioprótese vascular preservada em L-Hydro (Labcor Laboratórios^®^ ) é obtida pela aplicação de um processo de preservação conhecido como L-Hydro em artérias mesentéricas bovinas. A tecnologia L-Hydro permite a extração de antígenos e componentes celulares do tecido biológico ao mesmo tempo em que preserva constituintes extracelulares fundamentais, tais como elastina e colágeno. O comportamento biológico da bioprótese vascular preservada com tecnologia L-Hydro compartilha com a veia autógena a capacidade de alcançar a endotelização completa. A cicatrização do enxerto se completa com a incorporação de miofibroblastos ao arcabouço de colágeno e elastina na camada média da artéria mesentérica bovina 4 ^,^ 5 . 

 O objetivo deste estudo foi comparar a performance de FAVs confeccionadas com prótese vascular de ePTE com a daquelas que empregaram biopróteses vasculares preservadas com tecnologia L-Hydro, de acordo com os seguintes parâmetros: perviedade primária, perviedade primária assistida e perviedade secundária; propriedades de manuseio cirúrgico da prótese vascular utilizada na confecção da FAV; e complicações relacionadas à prótese vascular empregada na confecção da FAV. 

## MÉTODOS

 O protocolo da pesquisa foi avaliado pelo Comitê de Ética da instituição e registrado na Plataforma Brasil (número: 46166115.4.0000.5258). Todos os pacientes foram devidamente esclarecidos em relação à proposta de participação voluntária no estudo e assinaram o termo de consentimento previamente à avaliação pré-operatória. 

 Os critérios de inclusão no estudo foram: pacientes adultos portadores de insuficiência renal crônica dialítica sem veias nativas em membros superiores adequadas para confecção de FAV para hemodiálise; e diâmetro da artéria braquial > 3 mm na fossa cubital e da veia axilar > 3 mm em sua porção mais distal (segundo recomendação da Kidney Diseases Outcomes Quality Initiative, KDOQI), em medida realizada no pré-operatório por *duplex scan* . 

 Os pacientes foram divididos aleatoriamente em dois grupos: grupo FAV ePTFE, incluindo 10 pacientes que foram submetidos à confecção de FAV com prótese vascular de ePTFE (FlowLine Bipore vascular grafts, JOTEC^®^); e grupo FAV L-Hydro, incluindo 10 pacientes que foram submetidos à confecção de FAV com bioprótese vascular preservada com tecnologia L-Hydro (Labcor Laboratórios^®^ ). 

 Os pacientes foram prospectivamente incluídos no estudo de forma aleatória e alternadamente em cada um dos grupos de comparação, no período compreendido entre 01/08/13 e 31/12/15, totalizando 20 FAVs realizadas no Hospital Federal do Andaraí (HFA). 

 O procedimento cirúrgico foi realizado sob bloqueio anestésico de plexo braquial e sedação. Em todos os pacientes, a FAV foi confeccionada sob heparinização sistêmica, com o implante da prótese vascular (ePTFE ou L-Hydro) no plano subcutâneo na face anterolateral do braço (topografia do músculo bíceps). A anastomose proximal na artéria braquial foi realizada no nível da fossa cubital, e a anastomose distal, na porção mais distal da veia axilar. No grupo FAV L-Hydro, a anastomose na artéria braquial foi realizada antes da anastomose na veia axilar. A bioprótese foi preenchida com sangue arterial sob pressão, guiada através do túnel no plano subcutâneo, e exteriorizada no ponto de dissecção da veia axilar. Essa estratégia visou impedir torções e acotovelamento no trajeto da bioprótese no túnel subcutâneo. Todos os pacientes foram examinados precocemente no pós-operatório (ainda na sala de recuperação pós-anestésica) em relação à perfusão distal à FAV (tempo de enchimento capilar e força da mão ipsilateral) pelo cirurgião responsável pela confecção da FAV 6 . O tempo de seguimento pós-operatório do estudo foi de 2 anos. O protocolo de seguimento pós-operatório foi baseado nos critérios estabelecidos pela KDOQI 7 ( Figuras 1 e 2 ). 

**Figura 1:** Prótese antes do implante.

**Figura 2:** Prótese implantada.

 Todos os pacientes foram avaliados no seguimento pós-operatório por eco-Doppler vascular (*duplex scan*) da FAV para pesquisa de: estenose ou dilatação na prótese vascular; alterações na espessura da parede da prótese vascular; estenose no sítio das anastomoses (hiperplasia neointimal); presença de hematomas ou coleções peri-prótese; estimativa do fluxo da FAV 8 ; e avaliação da resposta à antibioticoterapia em caso de infecção da prótese ( Figura 3 ). 

**Figura 3:** Doppler de controle.

 Critérios clínicos da performance da FAV foram também avaliados durante as sessões de hemodiálise, tais como: presença de ondas de pulso no trajeto da FAV; presença de frêmito/sopro contínuo no trajeto da FAV; dificuldades encontradas na punção da prótese; detecção de sangramento persistente no local de punção após retirada da agulha; e necessidade de modificação de parâmetros na programação da máquina para atingir padrões de diálise efetiva 8 . 

### Oclusão aguda da FAV

 Em caso de suspeita clínica de oclusão da FAV (desaparecimento do frêmito ou sopro), a recomendação do protocolo do estudo foi a avaliação imediata do cirurgião e propedêutica com *duplex scan* ou angiografia. Após confirmação do diagnóstico, a intervenção terapêutica através de procedimento endovascular foi a conduta de escolha. Somente com a impossibilidade ou fracasso da terapêutica endovascular a abordagem cirúrgica direta foi realizada. 

### Infecção da FAV

 O diagnóstico de infecção da FAV foi estabelecido através de sinais clínicos (eritema, edema e dor no trajeto da prótese) e confirmado com hemocultura ou cultura de secreção. A antibioticoterapia prolongada foi o tratamento inicial proposto para infecção de FAV com prótese. A exérese cirúrgica da prótese vascular previamente implantada estava indicada em caso de falha do tratamento com antibióticos 9 . 

### Síndrome do roubo de fluxo

 A síndrome do roubo de fluxo foi definida segundo a classificação dos estágios de isquemia 9 ^,^ 10 :

- Estágio I: palidez da mão, que adquire tonalidade azul-claro e/ou hipotermia da mão, sem presença de dor;
- Estágio II: dor na mão desencadeada por exercício e/ou durante hemodiálise;
- Estágio III: dor de repouso na mão ipsilateral à FAV;
- Estágio IV: úlcera/necrose/gangrena na mão.

A intervenção cirúrgica estaria indicada em quadros de síndrome do roubo de fluxo classificados no estágio III ou IV. 

###  Técnica de punção da prótese para hemodiálise 

 A técnica de punção empregada para início da hemodiálise na FAV L-Hydro foi a mesma empregada em FAV com veias nativas: o primeiro acesso é realizado com agulhas de punção 17G em angulação de 25º a 30º. Após sessões subsequentes de hemodiálise, com a pele mais espessada nos locais de punção, a FAV L-Hydro passou a ser puncionada com uma agulha mais calibrosa (15G). Na FAV ePTFE, a técnica de punção da prótese utilizou a agulha com calibre 15G desde o primeiro acesso para hemodiálise 1 . 

No presente estudo, foram utilizadas as seguintes definições:

- Perviedade primária da FAV: definida como o intervalo de tempo decorrido entre a confecção da FAV e o diagnóstico de disfunção da FAV ou oclusão completa da prótese (trombose);
- Perviedade primária assistida da FAV: definida como o intervalo de tempo de durabilidade da FAV que sofreu intervenção (endovascular/cirurgia) realizada para corrigir disfunções diagnosticadas durante seguimento pós-operatório;
- Perviedade secundária da FAV: definida como o intervalo de tempo de durabilidade da FAV que sofreu intervenção (endovascular/cirurgia) realizada após oclusão completa da prótese (trombose).

O teste qui-quadrado foi utilizado para comparação das taxas de complicações encontradas no estudo. O nível de significância de probabilidade estatística utilizado no estudo foi de 0,05. 

## RESULTADOS

 Os dados demográficos dos pacientes dos grupos FAV L-Hydro e FAV ePTFE foram semelhantes. Em relação à frequência de comorbidades, o diagnóstico de hipertensão arterial sistêmica e intraoperatória foi mais frequente no grupo L-Hydro. 

 No seguimento pós-operatório, houve diferença entre os grupos, com um caso a mais no grupo FAV ePTFE (seis intervenções realizadas em três pacientes), enquanto no grupo FAV L-Hydro foram realizadas quatro intervenções em dois pacientes. 

 Em relação à perviedade primária assistida, os dados obtidos mostraram que 50% dos pacientes do grupo FAV L-Hydro apresentavam a fístula funcionante após 1 ano de seguimento e 25% após 2 anos de acompanhamento pós-operatório. No grupo FAV ePTFE, no primeiro ano de pós-operatório, 25% dos pacientes mostravam a fístula com função preservada e, após 2 anos, apenas 10% dos pacientes mantinham a FAV funcionante (p < 0,05). 

 Após 2 anos de seguimento, a perviedade primária no grupo FAV L-Hydro foi de 70%, e no grupo FAV ePTFE foi de somente 20% (p < 0,05). 

 A análise da perviedade secundária da FAV não revelou diferença estatística entre os grupos FAV L-Hydro e FAV ePTFE (perviedade secundária, após 2 anos de acompanhamento, de 60% e 50%, respectivamente). Na população estudada, o número de intervenções necessárias para prolongar a vida útil da FAV foi de 1,5 intervenção/ano no grupo FAV L-Hydro e de duas intervenções/ano no grupo FAV ePTFE. O procedimento mais frequentemente empregado para a preservação da função da FAV foi a angioplastia. 

 As complicações relacionadas à prótese vascular mais frequentemente observadas foram: trombose, infecção e pseudoaneurisma. A complicação mais frequente foi a trombose do enxerto, sendo observada em oito pacientes do grupo FAV ePTFE e três do grupo FAV L-Hydro (p = 0,01). A frequência de infecção de prótese foi semelhante em ambos os grupos estudados mas, no grupo FAV L-Hydro, diferentemente da conduta adotada no grupo FAV ePTFE, a terapia prolongada com antibióticos foi eficiente em evitar a necessidade de retirada da prótese infectada ( Figura 4 ). 

**Figura 4:** Complicações.

 A frequência do diagnóstico de outras complicações, como formação de pseudoaneurisma e síndrome do roubo de fluxo, não apresentou diferença estatística entre os dois grupos estudados. 

 Em um paciente do grupo FAV L-Hydro, com quadro de hipertensão venosa secundária à estenose de veia central, a bioprótese L-Hydro evoluiu com dilatação importante, motivando a desativação da FAV e retirada do enxerto. O exame anatomopatológico da prótese explantada mostrou integridade do tecido biológico, com revestimento endotelial preservado, sem sinais de degeneração. 

## DISCUSSÃO

 Apesar do número limitado de pacientes, observamos em nosso estudo maior perviedade primária da FAV confeccionada com a bioprótese L-Hydro, comparativamente à FAV com prótese de ePTFE 11 . Observamos também que, no grupo FAV L-Hydro, um menor número de intervenções foi necessário para manutenção da viabilidade da FAV. A menor necessidade de intervenções no grupo L-Hydro teve impacto na menor taxa de complicações encontrada nesse grupo 12 . 

 O conceito de perviedade primária da FAV está relacionado ao tempo decorrido entre a confecção da fístula e o diagnóstico de disfunção (ou oclusão). O diagnóstico de disfunção da FAV é geralmente suspeitado a partir de alterações clínicas, notadas pelo técnico ou enfermeiro, durante as sessões de hemodiálise. Os sinais e sintomas percebidos dependem da *expertise* e da experiência do profissional de hemodiálise que, em última análise, é o responsável pelo encaminhamento do paciente para a avaliação do cirurgião vascular. Como a perviedade primária da FAV é dependente do diagnóstico de disfunção da FAV, e esta última não pode ser padronizada em todos os pacientes, fica nítido que a utilização da variável perviedade primária para fins de comparação de performance da FAV fica comprometida. Sendo assim, consideramos que a perviedade primária assistida e a perviedade secundária se constituem como parâmetros mais sólidos para a comparação de performance da FAV entre os grupos do nosso estudo 13 . 

 Consideramos que os índices de perviedade primária assistida encontrados em nosso estudo no grupo FAV ePTFE 14 foram baixos (25% dos pacientes com FAV funcionante após 1 ano de seguimento), apesar de numerosos estudos prospectivos mostrarem taxas de perviedade primária assistida que variam de 10% a 43%. Dois estudos prospectivos recentes com FAV com enxertos de ePTFE 15 também relataram ampla discrepância de resultados. Em estudo retrospectivo comparando a performance de FAV confeccionadas com ePTFE *versus* PTFE padrão, enxertos de ePTFE 16 , revelaram taxas de perviedade primária assistida de 35% *versus* 25%, respectivamente. 

 Apesar da variabilidade de resultados encontrados em estudos comparativos de perviedade de FAV com próteses, é importante ressaltar o resultado encontrado em nosso estudo, que revela uma taxa de perviedade primária assistida de 70% no grupo FAV L-Hydro. O número limitado de pacientes incluídos em nossa amostra pode ter influenciado o resultado obtido. De todo modo, trata-se de resultado muito acima daquele observado na maioria dos estudos prospectivos publicados sobre perviedade primária assistida em FAV confeccionada com ePTFE. 

 Nossa experiência inicial com a bioprótese vascular preservada em L-Hydro começou em 2011 na revascularização de membros inferiores em pacientes sem veias nativas disponíveis e como opção à prótese vascular de ePTFE. Naquela ocasião, observamos menor resposta inflamatória no local do implante da bioprótese e menor taxa de complicações infecciosas, além de melhor maleabilidade cirúrgica do enxerto quando comparado à prótese vascular de ePTFE. Após acompanhamento pós-operatório médio de 15 meses desse grupo de pacientes, observamos uma incidência significativamente menor de trombose do enxerto, comparativamente às próteses vasculares de ePTFE 17 . 

 A hiperplasia neointimal que se desenvolve frequentemente nas anastomoses vasculares é causa de estenose progressiva e oclusão do enxerto. Uma causa comum de trombose do enxerto arteriovenoso é a hiperplasia neointimal que se desenvolve no local da anastomose. A causa da hiperplasia neointimal é o estresse mecânico multifatorial provocado pelo turbilhonamento do sangue na área da anastomose, pela diferença de complacência entre a parede arterial e a parede do substituto vascular e pelas forças de cisalhamento (“*shear stress*”) resultantes da alteração do fluxo sanguíneo na área da anastomose vascular. A maior complacência e maleabilidade da bioprótese vascular preservada em L-Hydro é uma evidência de que sua utilização para a confecção de FAVs resulta em um estímulo menor para a gênese da hiperplasia neointimal quando comparada à prótese vascular de ePTFE. 

## CONCLUSÃO

 O enxerto de artéria mesentérica bovina preservado em L-Hydro é uma excelente alternativa para hemodiálise. Estudos adicionais com número maior de participantes e maior tempo de seguimento pós-operatório são necessários para consolidação dos nossos resultados.
